# Gustatory Perception and Fat Body Energy Metabolism Are Jointly Affected by Vitellogenin and Juvenile Hormone in Honey Bees

**DOI:** 10.1371/journal.pgen.1002779

**Published:** 2012-06-28

**Authors:** Ying Wang, Colin S. Brent, Erin Fennern, Gro V. Amdam

**Affiliations:** 1 School of Life Sciences, Arizona State University, Tempe, Arizona, United States of America; 2 U.S. Department of Agriculture, Arid-Land Agricultural Research Center, Maricopa, Arizona, United States of America; 3 School of Medicine, Oregon Health and Science University, Portland, Oregon, United States of America; 4 Department of Chemistry, Biotechnology, and Food Science, University of Life Sciences, Aas, Norway; University of California San Francisco, United States of America

## Abstract

Honey bees (*Apis mellifera*) provide a system for studying social and food-related behavior. A caste of workers performs age-related tasks: young bees (nurses) usually feed the brood and other adult bees inside the nest, while older bees (foragers) forage outside for pollen, a protein/lipid source, or nectar, a carbohydrate source. The workers' transition from nursing to foraging and their foraging preferences correlate with differences in gustatory perception, metabolic gene expression, and endocrine physiology including the endocrine factors vitellogenin (Vg) and juvenile hormone (JH). However, the understanding of connections among social behavior, energy metabolism, and endocrine factors is incomplete. We used RNA interference (RNAi) to perturb the gene network of Vg and JH to learn more about these connections through effects on gustation, gene transcripts, and physiology. The RNAi perturbation was achieved by single and double knockdown of the genes *ultraspiracle* (*usp*) and *vg*, which encode a putative JH receptor and Vg, respectively. The double knockdown enhanced gustatory perception and elevated hemolymph glucose, trehalose, and JH. We also observed transcriptional responses in insulin like peptide 1 (*ilp1*), the adipokinetic hormone receptor (*AKHR*), and cGMP-dependent protein kinase (*PKG*, or “foraging gene” *Amfor*). Our study demonstrates that the Vg–JH regulatory module controls changes in carbohydrate metabolism, but not lipid metabolism, when worker bees shift from nursing to foraging. The module is also placed upstream of *ilp1*, *AKHR*, and *PKG* for the first time. As insulin, adipokinetic hormone (AKH), and PKG pathways influence metabolism and gustation in many animals, we propose that honey bees have conserved pathways in carbohydrate metabolism and conserved connections between energy metabolism and gustatory perception. Thus, perhaps the bee can make general contributions to the understanding of food-related behavior and metabolic disorders.

## Author Summary

Communication between internal energetic state and taste perception helps animals control food uptake and maintain normal life functions. Honey bees provide an animal model for studies of food-related behavior, such as the role of taste sensitivity in choice-making between carbohydrate- and protein-rich foods (nectar versus pollen for honey bees). A young bee's taste sensitivity to sugar predicts when she begins foraging later in life and influences her choice of foods. Vitellogenin (Vg), a protein produced in the bee's fat cells, and juvenile hormone (JH) influence honey bee taste perception and food-related behavior. Vg and JH are connected by a feedback loop, and we perturbed this Vg–JH circuit using a double gene knockdown approach. In response, bees became more sensitive to sugar, had higher sugar levels in the blood, and died faster during starvation, while lipid levels remained constant. We identified that *insulin like peptide 1* (*ilp1*), the *adipokinetic hormone receptor* (*AKHR*), and *PKG* (the *foraging* gene) were altered in the bees' fat cells after the perturbation. Our study demonstrates a new role for the Vg–JH circuit in honey bee carbohydrate metabolism, and it places Vg–JH upstream of the three metabolic genes that have conserved roles in food-related behaviors and energy metabolism in many animals.

## Introduction

Honey bees (*Apis mellifera*), with their complex social structure, remarkably plastic physiology and well-studied food-related activities, provide a model for connections between behavior and metabolism. Honey bee workers are essentially sterile female helpers that perform different tasks based on their age. In the first 2–3 weeks of adult life, workers called nurses care for the brood and other nestmates, construct wax combs and clean the nest. In roughly their 4th week of life, workers go through a behavioral transition and begin foraging outside the nest [1]. As foragers, workers can bias food-collection toward proteins (pollen) or carbohydrates (nectar). The transition to foraging behavior is associated with changes in gustatory perception [2], food consumption, hormone levels [3]–[5] and expression of genes associated with nutrient sensitivity and metabolism in workers [6]–[8]. Therefore, studies of honey bee behavioral physiology and genetics may reveal information of general interest in food-related behavior.

Gustatory perception is a predictor of honey bee behaviors such as how quickly a worker transitions from nursing to foraging (i.e., her age at foraging onset) and her foraging bias toward nectar vs. pollen (sources of carbohydrate vs. protein/lipid) [9], [10]. A worker's gustatory perception, or responsiveness, can be quantified by a standard method that involves monitoring her reflex response to an ascending series of sucrose concentrations [11]. Workers that respond to low sucrose concentrations have high gustatory responsiveness, and they forage at younger ages and collect more pollen and less nectar than bees with low gustatory responsiveness [9]. Gustatory sensitivity can also be an indicator of the bee's energy status: a worker bee with higher gustatory responsiveness is hungrier [12], and consumes more food (B. Rascon, G.V. Amdam, unpublished data) and dies faster under starvation conditions (M. Speth, G.V. Amdam, unpublished data) compared to a worker with lower gustatory responsiveness.

The social behaviors of honey bees are associated with complex energetic physiologies, suggesting that food consumption and food-related behavior are linked to energy homeostasis. Nurse bees, which feed on pollen and produce highly proteinaceous food secretions, have more abdominally stored proteins [13], [14] and lipids than foragers [15]. Experimental depletion of these stores triggers foraging behavior [16], [17]. The expression of insulin pathway genes [6] and the *adipokinetic hormone* (*AKH*) gene [18] also differs between the two behavioral stages. Similar differences are found between foragers with different food preferences: compared to nectar foragers, pollen foragers have higher *3-phosphoinositide-dependent kinase 1* (*PDK1*) transcript levels in the fat body (a functional homolog to the liver and white fat of vertebrates) [19]. PDK1 is a central kinase in the conserved insulin/insulin-like, epidermal growth factor, and target of rapamycin (TOR) nutrient signaling pathways [20], and *PDK1* has been genetically linked to foraging behavior [21]. Moreover, down-regulation of the *insulin receptor substrate* (*IRS*) gene in the fat body encourages foragers to collect more pollen and less nectar. This result provides a causal link between nutrient sensing and foraging preference in honey bees [8].

Established explanatory models of the nurse to forager transition of honey bees focus on the endocrine factors juvenile hormone (JH) and vitellogenin (Vg). JH is secreted from paired *corpora allata* neurohemal organs posterior to the honey bee brain [22], and affects development, maturation, and social behavior. Topical application of JH on nurse bees increases their gustatory responsiveness and causes an early transition to foraging [3], [4], [23]. Vg is a yolk protein precursor produced by the fat body [24], [25]. Vg has several functions in workers, including immune responses [26], oxidative stress resistance [27], and the production of proteinaceous secretions by nurses [28]. Vg operates in a feedback loop with JH, and appears to slow foraging onset by suppressing JH titer [16]. Down-regulation of Vg in nurse bees increases JH levels [29], enhances gustatory responsiveness [30], accelerates onset of foraging and encourages bees to collect nectar [31].

JH and Vg covary with energy metabolism in several insect species. In the fruit fly *Drosophila melanogaster* and the mosquito *Aedes aegypti*, insulin/insulin-like signaling is a major metabolic regulator that influences the production of yolk proteins like Vg [32]–[35] as well as JH synthesis [36]. Changing JH levels can in turn influence many metabolic processes (reviewed by Flatt et al. [37]). In honey bees and mosquitoes, Vg synthesis is enhanced by protein consumption [38] and inhibited by experimental interference of *TOR* [39], [40], a key energy sensing molecule (reviewed by Neufeld [41]). The TOR pathway crosstalks with insulin/insulin-like signaling [34] and is upstream of JH in honey bees [42].

Many studies, therefore, suggest connections between energy metabolism and nutrient pathways involving Vg and JH that can influence food-related behavior in honey bees (reviewed by Ament et al. [43]). However, it is unclear whether and how these connections are causal. For example, *vg* gene expression is influenced by *TOR* [40], but does not respond to knockdown of *IRS*, a central component of insulin/insulin-like signaling [8]. Depletion of JH by surgical removal of the *corpora allata*, furthermore, does not block foraging behavior, but can decrease flight muscle metabolic rate and alter worker flight behavior [44]. Overall, very few experiments have perturbed both Vg and JH. Such experiments can clarify connections between these factors, as well as implication for energy metabolism and food-related behavior.

Here, we manipulated *vg* and a putative receptor to JH separately and simultaneously in honey bee workers. We monitored the behavioral predictor, gustatory responsiveness, as well as starvation resistance; tested hemolymph carbohydrates and fat body lipid levels, and screened fat body tissue for expression of central metabolic genes. We predicted that gustatory perception, and carbohydrate and lipid metabolism would be influenced by the Vg-JH regulatory module. Manipulation of these two factors was achieved by knocking down *vg* and *ultraspiracle* (*usp*) separately and simultaneously in the fat body. *Vg* is the only Vg-encoding gene in honey bees [45], while the *usp* gene product (Usp) is a nuclear hormone receptor intimately involved in JH signaling and a strong candidate for a JH receptor [46]–[48]. Although there is more cumulative evidence for methoprene tolerant (Met) being a JH receptor in *Drosophila*, the honey bee *usp* gene has a documented function in JH signaling [49], [50], and was used here because JH (a terpenoid compound) cannot currently be targeted directly by gene knockdown.

Our data show that the double knockdown of *vg* and *usp* causes a mobilization of carbohydrates without changing the amount of stored lipid in worker bees. This result suggests a specific regulatory role of the Vg-JH module during the transition from nursing to foraging in workers. Conserved metabolic pathways (insulin, AKH and PKG) are influenced by the module, and we propose they may link energy metabolism to gustatory perception in honey bees like they do in many other animals.

## Results

### Validation of RNA interference (RNAi)–mediated gene knockdown

Wild-type newly emerged worker bees (<24 h old) injected with double stranded RNA (dsRNA) against *vg*, *usp* or both (double knockdown) were contrasted to workers injected with an established control solution of *gfp* dsRNA [30], [51]–[53]. The *gfp* gene encodes a green fluorescent protein (GFP) that is not present in bee genomes. Honey bee gene knockdown protocols are typically tested in the laboratory [27], [29], [54], where the JH response to *vg* knockdown was also previously observed [29], [54]. Consequently, knockdown and control bees were kept under laboratory conditions to verify knockdown and measure JH.

**Figure 1:**
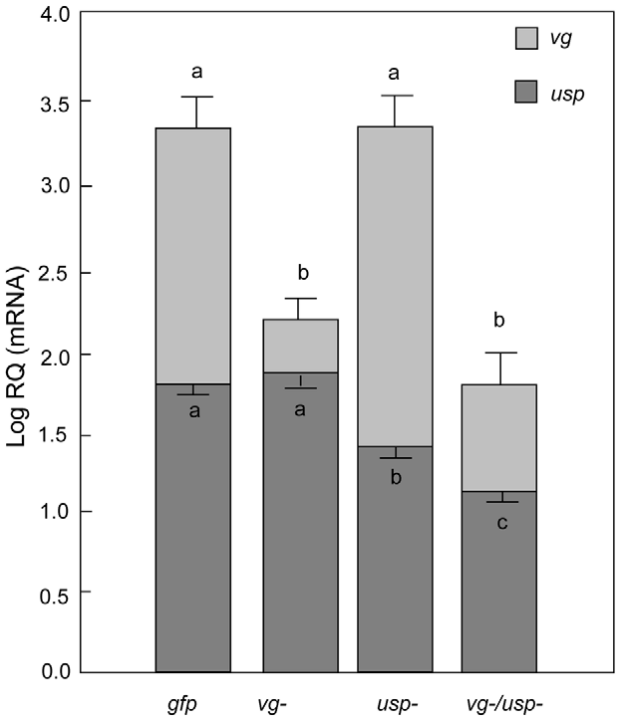
Single and double gene knockdown mediated by RNAi. For validation, RNA was obtained from the fat body tissue of 7-day old bees (Mean ± s.e., n = 21–22). The abbreviations *vg-*, *usp-*, *vg-/usp-* and *gfp* indicate *vg* single knockdown, *usp* single knockdown, *vg* and *usp* double knockdown, and *gfp* control, respectively. By *vg* RNAi, *vg* expression was significantly reduced without a change in *usp*. By *usp* RNAi, *usp* was significantly reduced without change in *vg*. Both *vg* and *usp* transcripts were reduced by *vg* and *usp* double knockdown and the reduction of *usp* in double knockdowns was bigger than with the single *usp*. Different letters above bars denote significant differences between treatment groups (p<0.05).

Six days after treatments, *vg* and *usp* transcript abundances were measured in individual samples of fat body (n = 21–22), which is the most accessible tissue to dsRNA in adult honey bees [55]. Both *vg* and *usp* had been significantly down-regulated by RNAi (Factorial ANOVA, *vg* mRNA: F*_vg-_*_ (1, 83)_ = 47.8701, p<0.0001; F*_usp-_*_ (1, 83)_ = 1.1494, p = 0.2868; and *usp* mRNA: F*_vg-_*_ (1, 83)_ = 3.117, p = 0.0812; F*_usp-_*_ (1, 83)_ = 40.603, p<0.0001). There was no significant interaction between the *vg* and *usp* knockdown treatments with regard to *vg* gene expression (Factorial ANOVA, *vg* RNA: F*_vg-_*_ & *usp-* (1, 83)_ = 0.9915, p = 0.3223), whereas significant interaction between *vg* and *usp* RNAi on *usp* transcript abundance suggests that the effect of *usp* RNAi on *usp* expression was strengthened by simultaneous *vg* RNAi (Factorial ANOVA, *usp* mRNA: F*_vg-_*_ & *usp-* (1, 83)_ = 6.1010, p = 0.0155). Post-hoc analyses revealed that *vg* single knockdowns had significantly reduced *vg* transcript levels (Fisher LSD: p _(*vg-* vs. *gfp*)_<0.0001), while the level of *usp* mRNA did not change (Fisher LSD: p _(*vg-* vs. *gfp*)_ = 0.6217) (Figure 1). Similarly, *usp* single knockdowns had reduced *usp* expression (Fisher LSD: p _(*usp-* vs. *gfp*)_<0.0001), while *vg* remained unchanged (Fisher LSD: p _(*vg-* vs. *gfp*)_ = 0.9573). The double knockdown treatment caused down-regulation of both *vg* and *usp* (Fisher LSD, *vg* mRNA: p _(*vg-/usp-* vs. *gfp*)_<0.0001; for *usp* mRNA: p _(*vg-/usp-* vs. *gfp*)_<0.0001) (Figure 1). The expression level of *usp*, moreover, was significantly lower in double knockdowns than in *usp* single knockdowns (Fisher LSD: p _(*vg-/usp-* vs. *usp-*)_ = 0.0034), while the *vg* mRNA level did not differ between the *vg* single knockdown and double knockdown groups (Fisher LSD: p _(*vg-/usp-* vs. *usp-*)_ = 0.1451). These results validated our *vg* and *usp* knockdowns, and showed that double knockdowns experienced stronger suppression of *usp* than *usp* knockdowns. *Usp* RNAi efficacy, therefore, may be enhanced when *vg* and *usp* are targeted together (Figure 1).

### Hemolymph JH titer

**Figure 2:**
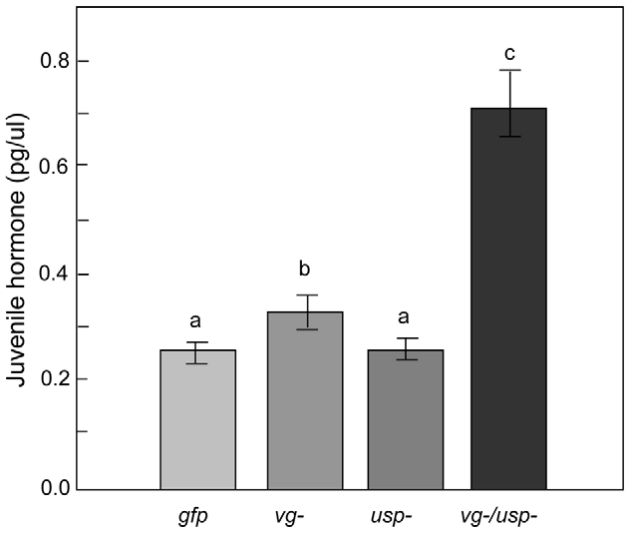
Gene knockdown effect on hemolymph JH titer. The circulating JH level significantly increased in *vg* single knockdown bees, as well as in *vg* and *usp* double knockdowns (Mean ± s.e., n = 13–16). Double knockdowns (*vg-/usp-*) had the highest JH titers among all treatment groups (p<0.05).

The workers' JH titer was significantly affected by both *vg* and *usp* RNAi when summing over the entire dataset from knockdowns and controls (Factorial ANOVA: F*_vg-_*_ (1, 55)_ = 8.0825, p = 0.0063; F*_usp-_*_ (1, 55)_ = 8.0825, p = 0.0429; n = 13–16). There was also a significant interaction effect between the *vg* and *usp* knockdown treatments on JH (Factorial ANOVA: F*_vg-_*_ & *usp-* (1, 55)_ = 4.7481, p = 0.0336). A post-hoc analysis revealed that the double knockdown caused a substantial increase in JH (Figure 2; Fisher LSD: p _(*vg-/usp-* vs. *gfp*)_ = 0.0004, p _(*vg-/usp-* vs. *usp-*)_ = 0.0008, p _(*vg-/usp-* vs. *vg-*)_ = 0.0024). In contrast, the separate knockdowns of *vg* and *usp* appeared to not affect the JH titer (Fisher LSD: p _(*vg-* vs. *gfp*)_ = 0.5524, p _(*vg-* vs. *usp-*)_ = 0.5950, p _(*usp-* vs. *gfp*)_ = 0.9751, p _(*usp-* vs. *vg-*)_ = 0.5950).

It was previously shown that *vg* knockdown can increase the JH level in honey bees [29], [54]. Therefore, we also did a targeted analysis to evaluate whether this effect could be visible in our data. We used a Student's t-test to compare the JH level of the *vg* and *usp* single knockdowns with that of the *gfp* controls. The test suggested that *usp* RNAi did not affect JH (Student's t-test: t _(1,27)_ = 0.0705, p = 0.9443), while *vg* RNAi caused a significant increase in the hormone titer (Student's t-test: t _(1, 25)_ = −0.7512, p = 0.0461). This response to *vg* down-regulation supports the repeatability of previous experiments and the hypothesis that *vg* can suppress JH [29], [54]. The significant interaction we detected between the *vg* and *usp* knockdown treatments further suggests that the release of JH which follows after *vg* knockdown becomes enhanced by simultaneous *usp* RNAi.

### Sucrose responsiveness

After the initial validation of RNAi efficacy in the laboratory, we moved forward to testing gustatory responsiveness, metabolic biology, physiology, and gene expression in the field. Natural honey bee colonies were preferred for these experiments since the sensory and metabolic traits of workers can be sensitive to social and nutritional factors that are difficult to fully account for in the laboratory [6], [56]. Single and double gene knockdowns were prepared like before, and the workers were transferred to colonies after dsRNA injection.

**Figure 3:**
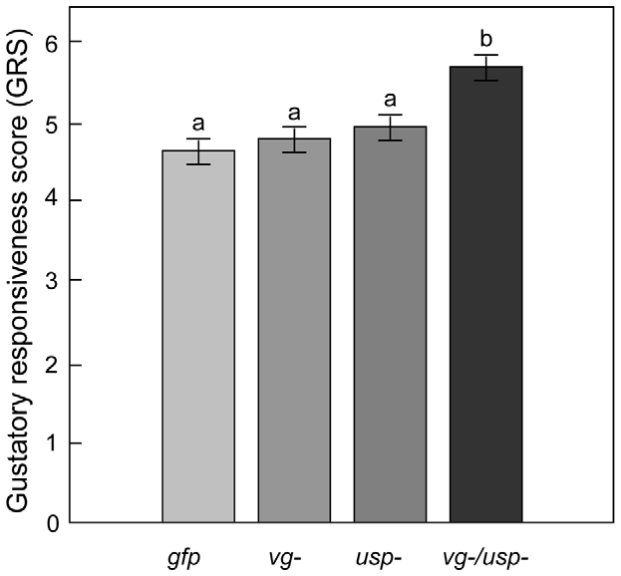
Gene knockdown effect on sucrose responsiveness in 7-day-old bees. The gustatory responsiveness score (GRS) was measured in the laboratory using the bees' proboscis extension response (PER). High GRS shows that bees responded to low sucrose concentrations, indicating a high gustatory sensitivity. Double knockdown bees (*vg-/usp-*) showed significantly increased gustatory responsiveness (Mean ± s.e., n = 33–65, p<0.05).

After six days, the knockdowns (*vg-*, *usp-*, *vg-/usp-*) and controls (*gfp*) were retrieved and exposed to water and a series of sucrose concentrations in the laboratory to measure their proboscis extension response (PER) [57], [58]. Individual bees were assigned a gustatory response score (GRS) based on a standard protocol [59]. We found the main effects of *vg* and *usp* RNAi had significant impact on GRS (Factorial ANOVA: F*_vg-_*_(1, 207)_ = 3.879, p = 0.0497; F*_usp-_*_ (1, 207)_ = 6.695, p = 0.0104; n = 33–65). Unlike the JH results, there was no interaction effect between *vg* and *usp* RNAi on GRS (Factorial ANOVA: F*_vg-_*_ & *usp-* (1, 207)_ = 1.829, p = 0.1777). Our post-hoc analysis (Figure 3) revealed that the GRSs of the *vg* and *usp* single knockdowns were similar to that of controls (Fisher LSD: p _(*vg-* vs. *gfp*)_ = 0.6296 and p _(*usp-* vs. *gfp*)_ = 0.3315). The double knockdown workers, on the other hand, had elevated GRSs (Fisher LSD: p _(*vg-/usp-* vs. *gfp*)_ = 0.0027, p _(*vg-/usp-* vs. *usp-*)_ = 0.0310, p _(*vg-/usp-* vs. *vg-*)_ = 0.0116). These results show that honey bee gustatory responsiveness is heightened when *vg* and *usp* are knocked down together, and suggest a joint effect in the regulation of this sensory modality by Vg and JH.

### Starvation resistance

To obtain a relative measure of the workers' metabolic reserves [60], we quantified their starvation resistance immediately after the GRS assay. Mortality was recorded every 3 hours.

**Figure 4:**
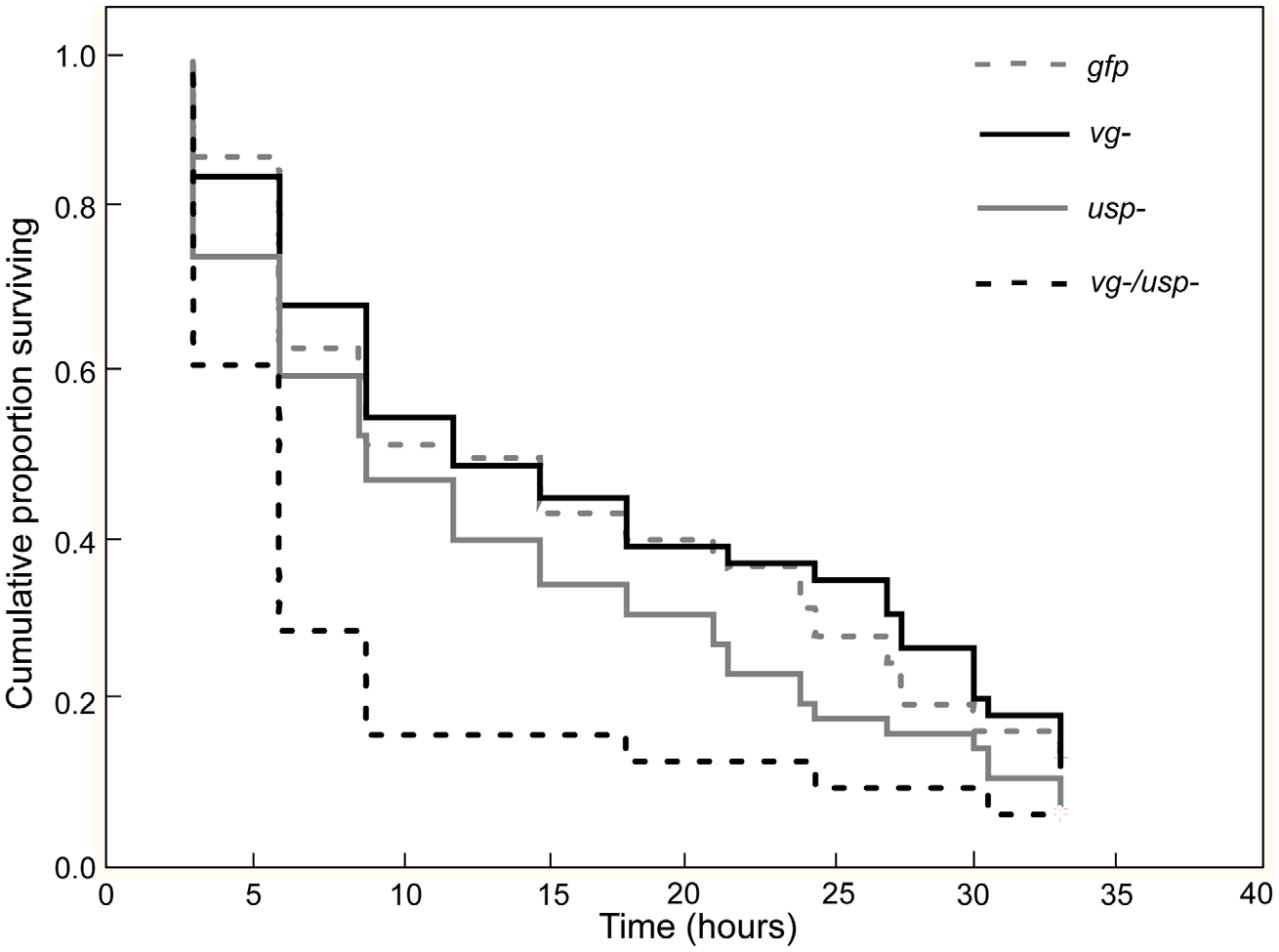
Gene knockdown effect on starvation resistance. After quantification of gustatory responsiveness (Figure 3), the bees were monitored for 3 days under starvation stress. Survival was recorded every 3 h. The single knockdown of the *vg* or *usp* gene did not affect the survival of the worker bees, whereas double knockdown (*vg*/*usp*) significantly shortened worker life span (n = 31–60, p<0.05).

Survival during starvation was significantly affected by RNAi (Figure 4; Chi-square = 14.1060, df = 3, p = 0.0028, n = 31–60). The double knockdown bees survived significantly shorter than the controls and single knockdown groups (Cox's F-Test: p _(*vg-/usp*- vs. *gfp*)_ = 0.0033, p _(*vg-/usp-* vs. *vg-*)_ = 0.0022 and p _(*vg-/usp-* vs. *usp-*)_ = 0.0405), while the survival of single knockdowns and controls was not statistically different (Cox's F-Test: P _(*vg-* vs. *gfp*)_ = 0.3532 and p _(*usp-* vs. *gfp*)_ = 0.0968). These findings suggest that the *vg* and *usp* double knockdown phenotype has a different metabolic biology than the other treatment groups. Such differences might include an increased mobilization rate and/or reduced amounts of metabolic reserves like circulating carbohydrates [61], [62] and abdominal lipid stores in the double knockdowns, causing them to be more susceptible to starvation.

### Circulating carbohydrates in hemolymph and lipid reserves in fat body

**Figure 5:**
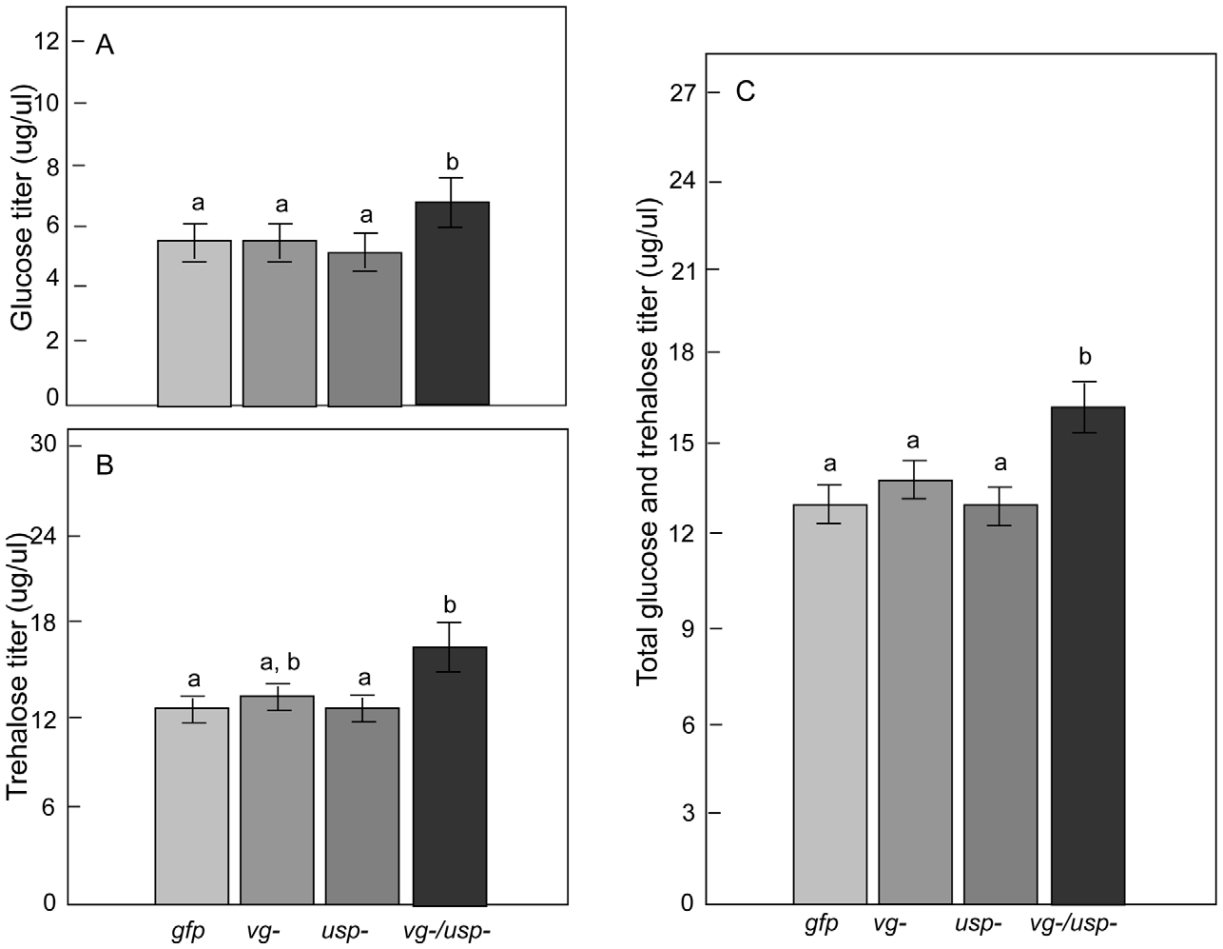
Gene knockdown effect on hemolymph glucose and trehalose. Hemolymph titers of (A) glucose, (B) trehalose and (C) combined glucose and trehalose. The titers in *vg* or *usp* single gene knockdown bees did not change compared to *gfp* control bees. In double knockdown bees (*vg*/*usp*), the glucose titer was significantly increased (Mean ± s.e., n  = 21–23, p<0.05), the trehalose titer showed a tentative increase (p = 0.0566) and the cumulative titer of glucose and trehalose was significantly elevated (p<0.05).

**Figure 6:**
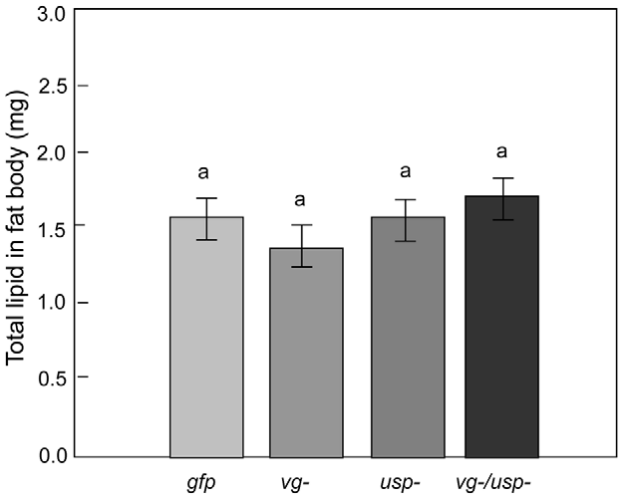
Gene knockdown effect on fat body lipid reserves. Abdominal lipid content was measured in the same bees that were used to measure hemolymph carbohydrate levels. Lipid content was not affected by the single or double knockdown of *vg* and *usp* (Mean ± s.e., n = 18–19, p<0.05).

We next measured the bees' blood (hemolymph) levels of glucose and trehalose [61] and the amount of lipids in their fat bodies to obtain more detailed information about their metabolic reserves. We found a significant main effect of *vg* RNAi on the glucose and trehalose titers of the bees (n = 21–23) (Figure 5A–5C; Factorial ANOVA: glucose, F*_vg-_*_ (1, 74)_ = 4.4310, p = 0.0387; F*_usp-_*_ (1, 74),_ = 2.0672, p = 0.1547; F*_vg-_*_ & *usp-* (1, 74),_ = 3.5384, p = 0.0639; trehalose, F*_vg-_*_ (1, 74)_ = 5.6163, p = 0.0204; F*_usp-_*_ (1, 74),_ = 1.1438, p = 0.2883; F *_vg-_*_ & *usp-* (1, 74),_ = 1.0946, p = 0.2989). In contrast, the fat body lipid content was unchanged by RNAi (Figure 6; Factorial ANOVA: F*_vg-_*_ (1, 60),_ = 0.2328, p = 0.6312; F*_usp-_*_ (1, 60),_ = 0.2447, p = 0.6226; F *_vg-_*_ & *usp-* (1, 60),_ = 2.0314, p = 0.1593; n = 18–19).

A post-hoc analysis revealed that double knockdowns had a higher glucose titer than the controls and both single knockdowns (Fisher LSD: p _(*vg-/usp-* vs. *gfp*)_ = 0.0241, p _(*vg-/usp-* vs. *vg-*)_ = 0.0245, p _(*vg-/usp-* vs. *usp-*)_ = 0.0106), as well as a higher trehalose titer than the controls and the *usp* single knockdowns (Fisher LSD: p _(*vg-/usp-* vs. *gfp*)_ = 0.0262, p _(*vg-/usp-* vs. *usp-*)_ = 0.0219) (Figure 5). Neither of the single knockdowns significantly affected the glucose (Fisher LSD: p _(*vg-* vs. *gfp*)_ = 0.9451 and p _(*usp-* vs. *gfp*)_ = 0.8224) or trehalose titer (Fisher LSD: p _(*vg-* vs. *gfp*)_ = 0.4495 and p _(*usp-* vs. *gfp*)_ = 0.9661). A similar pattern held when using a combined measure of major sugar contents in the blood, with significant main effect of *vg* RNAi (Factorial ANOVA: F*_vg-_*_ (1, 74)_ = 7.4914, p = 0.0078; F*_usp-_*_ (1, 74),_ = 2.1354, p = 0.1482; F*_vg-_*_ & *usp-* (1, 74),_ = 2.7208, p = 0.1033; n = 21–23) and the strongest influence of the double knockdown (Post-hoc, Fisher LSD: p _(*vg-/usp-* vs. *gfp*)_ = 0.0074, p _(*vg-/usp-* vs. *vg-*)_ = 0.0304, p _(*vg-/usp-* vs. *usp-*)_ = 0.0042).

Collectively these findings suggest that the combined knockdown of *vg* and *usp* has consequences for worker carbohydrate metabolism, measured as the mobilization of the major sugars to the blood. It is possible that *vg* is the main driver of these changes since *usp* manipulation had no discernable effects in single knockdowns. The effect of *vg* RNAi on circulating carbohydrate levels would thus be enhanced by *usp* RNAi in honey bees.

### Responses in associated gene networks

The fat body is the primary storage organ for metabolic reserves in bees and most insects in general [63]. To evaluate how relevant gene networks in this tissue responded to *vg* and *usp* knockdown, we did a targeted expression test of *insulin like peptide 1* (*ilp1*) and *2* (*ilp2*), which encode the proposed ligands of the insulin receptors of honey bees [6]. Additional measures were taken of *cGMP-dependent protein kinase* (*PKG*, also called the ‘foraging gene’ or *Amfor*), *JH esterase* (*JHE*), *adipokinetic hormone* gene (*AKH*) and its receptor (*AKHR*). *PKG* is associated with feeding behavior [64] and gustatory responsiveness [65] in *Drosophila*, division of labor in honey bees [66], [67], as well as energy metabolism and food intake in vertebrates [68], [69]. Expression of *JHE*, which encodes the primary JH-degrading enzyme in honey bees [52], was measured to determine whether the elevated JH titers in double knockdowns were associated with this gene. *AKH* (JN983824) and *AKHR* play conserved roles in glucose and lipid homeostasis. *AKH* encodes a peptide hormone (adipokinetic hormone, AKH), which is a glucagon analog primarily secreted by the *corpora cardiaca* (CC) complex at the base of the honey bee brain. We therefore examined levels of *AKH* and *AKHR* in the head capsule in addition to levels in the fat body.

**Figure 7:**
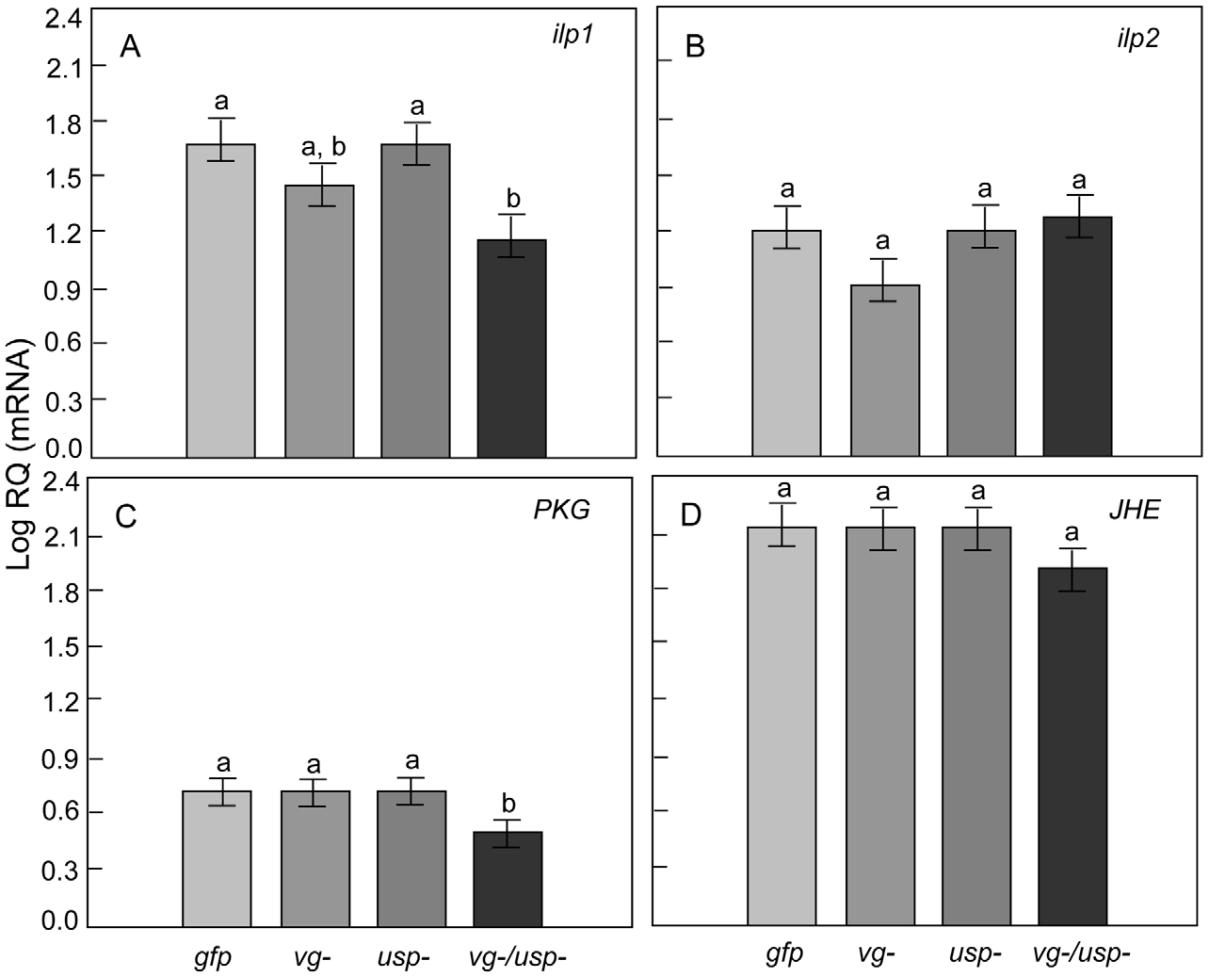
Gene knockdown effect on metabolically associated genes in the fat body. Relative expression of (A) *Insulin-like peptide 1* (*ilp1*), (B) *Insulin-like peptide 2* (*ilp2*), (C) *cGMP-dependent protein kinase* (*PKG*) and (D) *Juvenile hormone esterase* (*JHE*). The double knockdown of *vg* and *usp* resulted in reduced *ilp1* and *PKG* transcripts levels (Mean ± s.e., n = 16, p<0.05).

We found a significant main effect of *vg* RNAi on *ilp1* (Factorial ANOVA: F*_vg-_*_ (1, 60)_ = 5.3938, p = 0.0236), while its gene expression remained unchanged after *usp* RNAi (F*_usp-_*_ (1,60)_ = 1.0064, p = 0.3198; F*_vg-_*_ & *usp-* (1, 60),_ = 0.1744, p = 0.6777; n = 16). Compared to single knockdowns and controls, the double knockdown bees expressed less *ilp1* (Figure 7; post-hoc, Fisher LSD: p _(*vg-/usp-* vs. *gfp*)_ = 0.0219, p _(*vg-/usp-* vs. *vg-*)_ = 0.3191, p _(*vg-/usp-* vs. *usp-*)_ = 0.0574). The main effect of *vg* RNAi had no significant influence on *PKG* expression, while *usp* RNAi showed a tendency to reduce *PKG* (Factorial ANOVA: F*_vg-_*_ (1, 53)_ = 1.7569, p = 0.1907; F*_usp-_*_ (1,53)_ = 2.8860, p = 0.0952; F*_vg-_*_ & *usp-* (1, 53),_ = 2.3039, p = 0.1350; n = 16). Our post-hoc analysis revealed that *PKG* mRNA levels were significantly reduced in double knockdowns (Figure 7; Fisher LSD, p _(*vg-/usp-* vs. *gfp*)_ = 0.0389, p _(*vg-/usp-* vs. *vg-*)_ = 0.0348, p _(*vg-/usp-* vs. *usp-*)_ = 0.0582). In contrast, none of the RNAi treatments affected the expression of either *ilp2* (Factorial ANOVA: F*_vg-_*_ (1, 58)_ = 0.4949, p = 0.4846; F*_usp-_*_ (1,58)_ = 1.8074, p = 0.1841; F*_vg-_*_ & *usp-* (1, 58),_ = 1.1661, p = 0.2847; n = 16)(Figure 7) or *JHE* (Factorial ANOVA: F*_vg-_*_ (1, 60)_ = 0.4528, p = 0.5036; F*_usp-_*_ (1,60)_ = 0.1323, p = 0.7173; F*_vg-_*_ & *usp-* (1, 60),_ = 0.17460, p = 0.6763; n = 16) (Figure 7).

**Figure 8:**
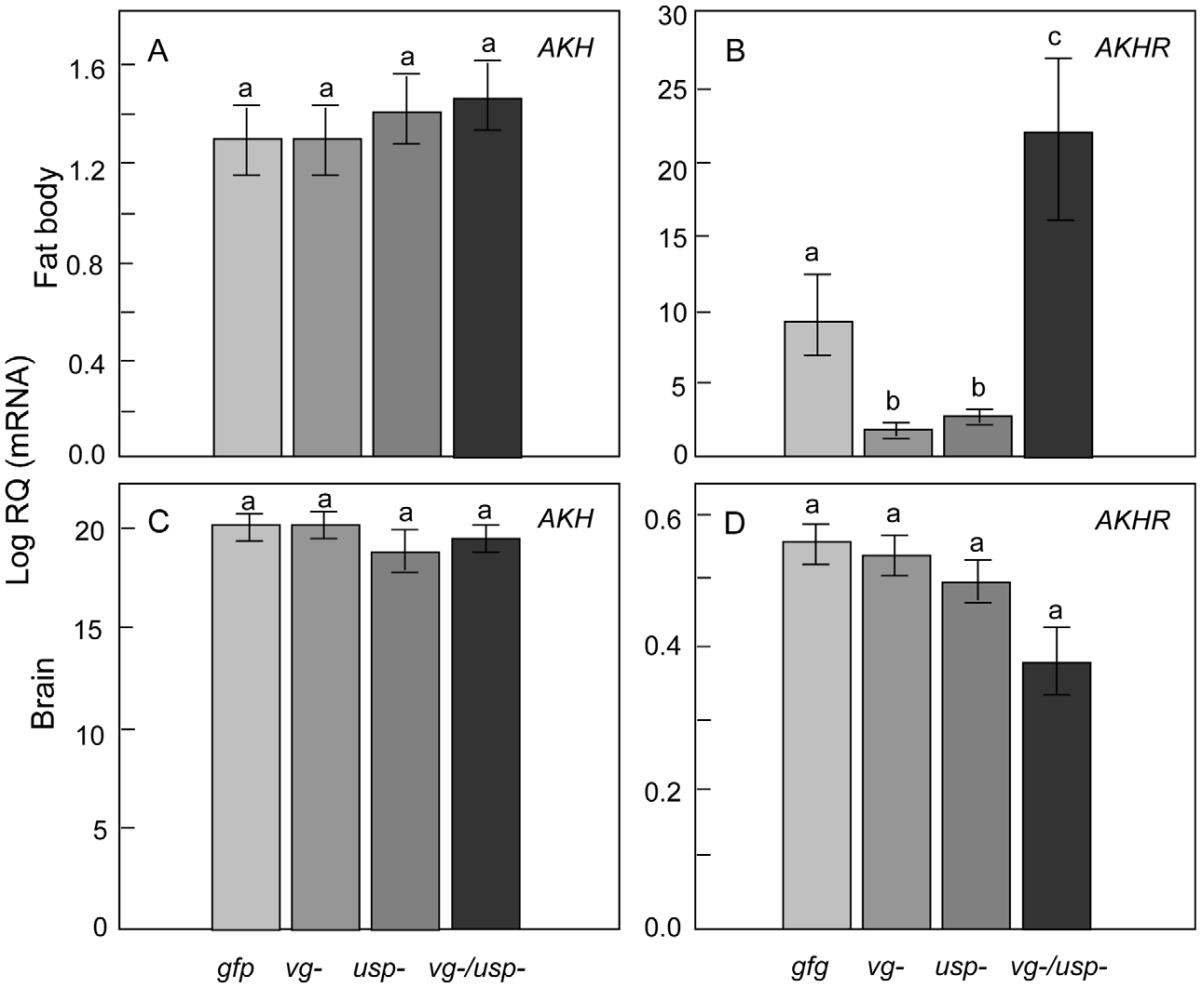
Gene knockdown effect on *adipokinetic hormone* (*AKH*) and *adipokinetic hormone receptor* (*AKHR*) genes in worker fat body and head. Relative expression of (A) *AKH*, (B) *AKHR* in the fat body, and (C) *AKH*, (D) *AKHR* in the brain. While *AKH* remained unaffected, the double knockdowns of *vg* and *usp* had significantly elevated *AKHR* transcript levels in fat body compared to control. The *vg* and *usp* single knockdowns, in contrast, had lower levels of *AKHR* in the fat body (Mean ± s.e., n = 12, p<0.05).

Although honey bee *AKH* is a predicted gene (Genbank: GB30028-RA) [70], several *AKH* splice variants (likely with different functions) are verified in insects such as *Tribolium* [71] and *Bombyx* [72].We cloned this gene to learn its architecture in *A. mellifera*, and our analysis validated a single *AKH* preprohormone transcript (Figure S1, GenBank: JN983824), which we used in our expression screen. RNAi did not affect *AKH* in fat body (Figure 8: Factorial ANOVA: F*_vg-_*_ (1, 44)_ = 0.0342, p = 0.8542; F*_usp-_*_ (1, 44)_ = 0.1180, p = 0.7329; F*_vg-_*_ & *usp-* (1, 44),_ = 0.0219, p = 0.8829, n = 12) or head (Factorial ANOVA: F*_vg-_*_ (1, 43)_ = 0.0920, p = 0.7626; F*_usp-_*_ (1, 43)_ = 1.9350, p = 0.1714; F*_vg-_*_ & *usp-* (1, 43),_ =  0.1650, p = 0.6883; n = 11–12). However, we detected significant main effects of both *vg* and *usp* RNAi on fat body *AKHR* levels (Factorial ANOVA: F*_vg-_*_ (1, 44)_ = 6.7029, p = 0.0130; F*_usp-_*_ (1, 44)_ = 1.8.5670, p = 0.0054; n = 12). The interaction effect between *vg* and *usp* RNAi was also significant in the case of *AKHR* (Factorial ANOVA, F*_vg-_*_ & *usp-* (1, 44),_ = 34.3282, p<0.0001). Here, a post-hoc analysis showed *AKHR* was elevated in double knockdowns compared to single knockdowns and *gfp* controls (Fisher LSD, p _(*vg-/usp-* vs. *gfp*)_ = 0.0003, p _(*vg-/usp-* vs. *vg-*)_<0.0001, p _(*vg-/usp-* vs. *usp-*)_<0.0001), while *vg* (Fisher LSD, p _(*vg-* vs. *gfp*)_ = 0.0255, p _(*vg-* vs. *vg-/usp-*)_<0.0001) and *usp* single knockdowns (Fisher LSD, p _(*usp-* vs. *gfp*)_ = 0.0440, p _(*vg-* vs. *vg-/usp-*)_<0.0001) reduced *AKHR* compared to the controls and double knockdowns. Perhaps the negative effect that *vg* and *usp* RNAi each has on *AKHR* expression causes a compensatory response in the double knockdowns that otherwise would experience an additive suppression of the *AKHR* gene. Head levels of *AKHR* were not affected in our experiments (Figure 8; Factorial ANOVA: F*_vg-_*_ (1, 44)_ = 1.3263, p = 0.2557; F*_usp-_*_ (1, 44)_ = 2.0433, p = 0.1600; F*_vg-_*_ & *usp-* (1, 44),_ = 1.0529, p = 0.3105; n = 12), but double knockdowns showed a tentative reduction relative to controls (Fisher LSD, p _(*vg-/usp-* vs. *gfp*)_ = 0.0748).

In summary, our transcript screen determined that several genes relevant to metabolic biology responded in fat body after combinations of *vg* and *usp* knockdown. The strongest responses were detected after double gene knockdown.

## Discussion

Here, we used a single and double gene knockdown strategy to study how Vg and JH can affect the sensory, metabolic and behavioral biology of honey bee workers. Double gene knockdown was previously reported for honey bee larvae [42], while our work (this paper) represents the first successful protocol for adult bees. Suppression of the putative JH receptor *usp* did not induce measurable changes in sucrose responsiveness and worker physiology, while the suppression of *vg* caused a subtle but significant increase in JH (previously observed by Guidugli et al. [29]). The simultaneous knockdown of *vg* and *usp*, in contrast, increased sucrose sensitivity, reduced starvation resistance, heightened glucose, trehalose and JH in hemolymph, and altered the expression of a set of nutrient signaling genes in the fat body. The same amount of dsRNA was used in all our experimental treatments, which rules out the possibility that the effects of double knockdown represent a simple dose-response to the injected material.

Our results may contradict the previous finding that *vg* RNAi affects sucrose responsiveness [30]. These two experiments, however, were performed in different environments (Davis California vs. Tempe, Arizona, USA) with bees of different ages (5 vs. 7 days old). Environmental factors such as season, temperature and nectar availability can affect gustatory sensitivity, as does worker age [56]. Therefore, we cannot conclude that the studies are fully comparable. It is possible that *vg* RNAi has measurable sensory effects in some conditions and ages, but not others. In comparison, the simultaneous suppression of *vg* and *usp* had a dramatic effect on sucrose responsiveness. The outcome may be related to the strongly elevated JH level of the double knockdowns, since JH application can lead to elevated gustatory sensitivity [56]. Further experimentation is required to test this hypothesis.

Despite the open questions, our experiments were successful in perturbing a regulatory module of honey bee biology and behavior that relies on Vg and JH [16], [31]. This perturbation had consequences that inform about connections between Vg and JH, and about relationships between this module, metabolic biology, sucrose sensory perception and foraging behavior in honey bee workers.

### The relationship between Vg and JH in honey bee worker behavioral physiology

Our study targeted the Vg and JH feedback loop of honey bee workers that is central to the bees' transition from nursing to foraging. The basic ability of Vg and JH to mutually suppress each other was confirmed in previous experiments, but their exact relationship is poorly defined. We determined that Vg must reduce JH other than by accelerating its degradation given that levels of *JHE*, encoding the primary JH-degrading enzyme in honey bees [52], were unaffected by RNAi. Instead, our study suggests that Vg may inhibit JH production. This proposition can be tested in future studies focused on the regulation of JH synthesis. Moreover, we found a significant interaction effect of *vg* and *usp* RNAi on *usp* transcript abundance and the JH titer, which suggests that the increase in JH induced by *vg* RNAi is accelerated by simultaneous *usp* knockdown. Perhaps this result points to a compensatory response to a quantitative reduction in the JH receptor. Similar compensation is a common mechanism to overcome impaired function of receptors in mammals [73], [74]. Possibly, such compensation was not observed after *usp* single knockdown because *vg* remained at control levels in these bees, continuing to suppress JH.

Usp proteins are also binding partners of the ecdysone receptor (EcR) in insects (reviewed in [75]). Ecdysteroids were not monitored in our experiment, as titers are generally very low in adult worker bees and several studies suggest a loss-of-function of the ecdysteroids in adult eusocial insects [76]. Recent research, on the other hand, suggests that ecdysteroids can influence some aspects of bee behavior [19], [77] We cannot exclude that our *usp* knockdown affected such relationships, as they were not specifically tracked in the experiments.

### Metabolic biology of the nurse to forager transition

The metabolic biology of honey bee nurses and foragers differ. In nurses, the fat body is biased toward lipid and protein metabolism, whereas carbohydrate metabolism dominates the fat body of foragers [18]. Similarly, we observed a mobilization of sugars in double knockdowns, which are like foragers had an elevated level of JH [78] and an increased susceptibility to starvation [15]. Previous studies, moreover, have suggested that the AKH pathway is active in foragers [18], [43], and we observed an increase in *AKHR* expression in double knockdowns. Therefore, we believe that the simultaneous knockdown of *vg* and *usp* in worker honey bees provides an informative model for the role of the Vg-JH module in the nurse to forager transition.

What we have learned from testing this model is that although carbohydrate and lipid metabolisms change concurrently during the transition from nursing to foraging [17], [18], the regulation of the two systems can be decoupled. In our experiment, abdominal lipid stores were not significantly affected by double knockdown; these stores were equal between all treatment groups. This finding suggests that the regulation of lipid metabolism occurs largely independently of the Vg-JH relationship. In contrast, our results place carbohydrate metabolism downstream of the Vg-JH regulatory module. We propose, therefore, that the double knockdown phenotype, which diverged from the other treatment groups for many characteristics including starvation resistance, is not explained by altered lipid metabolism but by altered carbohydrate metabolism. We speculate that an increased carbohydrate metabolism driven by the Vg-JH module can be a central feature in the maturational development of honey bee foragers. It is generally known that worker bees experience a substantial lipid loss during the transition from nurse bee to forager, supporting a shift from lipid- to carbohydrate metabolism [18], [43].

### Responses in metabolic gene networks

Our results support previous studies that suggested roles of *ilp1, AKH* and *PKG* in the nurse to forager transition of honey bees [18], [79]. Insulin like peptides (ilps) play conserved roles in carbohydrate and amino acid metabolisms in vertebrates and invertebrates. In *Drosophila*, brain expression of *ilp*s (called *dilp* genes) regulates trehalose and glucose levels in the hemolymph [80]–[82]. Patterns of *ilp* expression in the brain and fat body have been suggested to explain nutritional status and behavioral transitions in worker honey bees [6], [54]. In fat body, *ilp1* correlates positively with *vg* when amino acids are available to worker bees [54], but it is not fully understood how ilps regulate worker energy metabolism and behavior. Our results suggest that *ilp1* is a nutrient sensor gene of the honey bee fat body that can be controlled by the Vg-JH module of worker bees. JH might up-regulate *ilps* produced by the fat body of *Tribolium* [83], but a previous study did not find a similar connection between JH and the expression of *ilp1* in honey bee fat body cell [54]. Thus, it is unclear how closely connected these endocrine factors are in honey bees.

Insect AKH is produced by the *corpora cardiaca*, paired neurosecretory organs connected to the JH producing *corpora allata*. As a functional analog of mammalian glucagon, AKH is responsible for mobilization of carbohydrates and lipids, and regulates the release of nutrients such as trehalose into hemolymph in *Drosophila melanogaster* [84], [85]. Genetic deletion of the AKH receptor gene (*AKHR*) produces obese and starvation resistant flies [84], [85], while *AKHR* overexpression induces mobilization of carbohydrates, but not lipids, in both *Drosophila* and *Manduca sexta* [86], [87]. In our study, double knockdown bees are characterized by elevated *AKHR* expression, starvation susceptibility and elevated carbohydrate levels in the blood, but no changes in fat body lipid stores. Typically, nursing honey bees have about 1.4–2.4 mg lipid per fat body [15], and our observations are within the same range (1.5–2.2 mg lipid per fat body). This finding of normal lipid stores in double knockdown bees suggests that the increased amounts of *AKHR* mRNA in the same animals are not linked to lipid mobilization. Instead, the overexpression of *AKHR* might be more exclusively associated with carbohydrate mobilization in honey bees. Since *AKH* expression remained unchanged in the head and fat body of double knockdowns, it is also unlikely that the increase in *AKHR* mRNA was paralleled by elevated AKH titers. Instead, it appears that fat body *AKHR* expression can be regulated by the Vg-JH module in worker bees, providing a candidate mechanism that may explain how the feedback loop propels workers from one physiological and behavioral state to the other [16]. Furthermore, the down-regulation of *AKHR* by both the *vg* and *usp* single knockdown supports placement of *AKHR* downstream of *vg* and *usp*, and highlights the complexity of *AKHR* regulation.

*PKG*, or *Amfor*, is associated with foraging behaviors [79], [88] in both *Drosophila* and honey bees. Studies on PKG mutant flies suggest that PKG is involved in carbohydrate metabolism and affects *AKH* expression [88]. However, whether *PKG* is involved in energy metabolism is incompletely understood in honey bees. We find that *PKG* expression covaries with changes in honey bee gustatory responsiveness and metabolic biology, and we provide the first evidence that places *PKG* downstream of the Vg-JH feedback relationship. Specifically, we find *PKG* and *ilp1* are down-regulated in the fat body in response to the simultaneous knockdown of *vg* and *usp*. This directional change is opposite to patterns seen in the brain, where an up-regulation has been linked to the transition from nursing to foraging behavior. Studies suggest the honey bee brain has metabolic patterns that are distinct from those of other tissues including the fat body (reviewed by Ament et al. [43]). The opposite expression patterns of *ilp1* and *PKG* in the brain [6], [66] and fat body (this study) exemplify such tissue specificity.

### Conserved carbohydrate pathways and conserved connection between gustatory perception and energy metabolism

Relative to control workers, the double knockdown bees had high blood levels of glucose and trehalose, normal fat body lipid stores, low amounts of insulin like peptide (*ilp1*) mRNA and high amounts of *AKHR* mRNA. Mammalian type 1 diabetes is similarly characterized by high blood glucose, the absence of obesity, reduced insulin production, and inadequate suppression of glucagon secretion. The parallels between these phenotypes may indicate that the regulatory system of carbohydrate metabolism has conserved features that are shared between honey bees and mammals. Similarities may also exist in the way that metabolic processes are linked to gustatory perception. In mammals, key metabolic regulators like leptin, insulin and glucagon [89], [90], also modulate sweet taste perception (reviewed by [91]) and taste sensitivity [92]. Although similar connections are not equally well understood in social insects, our study showed gustatory responsiveness changed substantially in response to *vg* and *usp* double knockdown, and this could be a result of strongly elevated JH levels. However, the metabolic genes *ilp1, AKHR* and *PKG* changed in parallel, and interestingly, their homologues influence gustatory perception in model animals like *D. melanogaster* and *Caenorhabditis elegans* [65], [84], [93]. Therefore, an alternative hypothesis is that *ilp1*, *AKHR* and *PKG* jointly affect gustatory perception and energy metabolism in honey bees. Should this prove to be the case, it would strengthen the similarities between insect and mammalian control systems, enhancing the utility and desirability of using honey bees to model basic mechanisms of carbohydrate metabolism, gustation and food-related behavior. These are areas of increasing importance in studies of human metabolic syndromes such as obesity and diabetes [94], [95]. Reciprocally, perhaps mammalian model systems can tell us more about metabolic biology that is important for bee health and pollination services.

## Materials and Methods

### Preparation of dsRNA

The *vg* and *usp* genes were partially cloned and used as templates in PCR. The PCR primers were established by previous studies [50], [96]. PCR products were purified using Qiaquick PCR purification kit (Qiagen, Frederick, MD, USA). DsRNA was synthesized using RiboMax Large Scale T7 RNA Production Systems (Promega, Madison, WI, USA) following the manufacturer's protocol. DsRNA toward green fluorescent protein (*gfp*) sequence, which is not found in bee genomes, was synthesized as a control from AF097553 template as before [96], [97]. DsRNA toward *vg*, *usp*, and *gfp* was purified using a phenol extraction. Aliquots were run on a 1% agarose gel for verification of dsRNA size and purity. For injections, the dsRNA was diluted to10 µg/µl in nuclease free water.

### Bees for validation of RNAi in the laboratory

Wild-type bees were maintained at the Honey Bee Research Laboratory at the Arizona State University Polytechnic Campus, Gilbert AZ. Equal numbers of newly emerged bees from nine wild-type colonies were mixed together, randomly assigned to four treatment groups and marked with enamel paint (Testors Corporation, Rockford, IL, USA). The bees were injected intra-abdominally with 20 ug dsRNA against either *gfp*, *vg* [31], *usp*, or both *vg* and *usp*. In total, about 50 bees were injected per group. Bees were held for six days in three two-compartment cages: on one side of a single wire-mesh screen were the treated bees, and on the other side were 200 presumed nurses that had been brushed from a comb of open brood cell containing larvae. This setup ensured that the experimental bees received normal social interactions and nourishment (Amdam et al., 2007). Bees in both compartments had access to a 30% sucrose solution and pollen dough (Crockett Honey, Tempe, AZ, USA). Fresh food was given daily. Cages were incubated at 33°C and 70% RH. When bees were 7-day old adults, their fat body and hemolymph were collected and flash-frozen in liquid nitrogen for *vg* and *usp* knockdown verification as well as JH titration.

### Quantification of *vg* and *usp* expression

To validate the single and double gene knockdowns, fat bodies were dissected from 7-day old marked bees, flash-frozen in liquid nitrogen, and stored at −80°C until use. The standard Trizol procedure (Promega, Madison, WI, USA) was used for RNA extraction. Isolated RNA was treated with DNaseI (Ambion, Austin, TX, USA) then expression levels were analyzed by a two-step qRT-PCR [19]. RNA was diluted to 200 ng/µl for the reverse transcription using TaqMan Reagents (Applied Biosystems, Foster City, CA, USA). Relative transcript abundance in each sample was measured in triplicates by real-time PCR (ABI Prism 7500, Applied Biosystems). Actin (GenBank:XM_623378) served as a reference gene because it has stable expressions in different honey bee tissues [98], [99] and is commonly used in gene expression studies in honey bees [100], [101]. The primer sequences are listed in table 1. Data were analyzed using the Delta-Delta CT method [102]. By monitoring negative control samples (without reverse transcriptase) and melting curves, we verified that the qRT-PCR assay was not confounded by DNA contamination or primer dimmers [103].

**Table 1:** Gene primers for real-time PCR.

Gene name	Forward primer	Reverse primer
*vg* (AJ517411)	GTTGGAGAGCAACATGCAGA	TCGATCCATTCCTTGATGGT
*usp* (GB16648)	GCGAAGAGAAATCCTGCATC	TCCCTTTCCTTGGTACGTTG
*JHE* (GB15327)	GGGTTGCCCTACACGTAATG	CGAACGGTGTGAATGGATTA
*ilp1* (GB17332-PA)	CGATAGTCCTGGTCGGTTTG	CAAGCTGAGCATAGCTGCAC
*ilp2* (GB10174-PA)	TAGGAGCGCAACTCCTCTGT	TTCCAGAAATGGAGATGGATG
*PKG* (GB18394)	AGTGAGTTGCCTGGTGATAG	TCGACGAGCTGTCTTTGTAT
*AKH* (JH983824)	CGTAAGCTTCGACCAAGTTTTT	CATTCGACAACTCCGATCCT
*AKHR* (GB16857)	ATAATCACCACCACGGGATT	GACCTTCGTTGAATCGCATA
*actin* (XM_623378)	TGCCAACACTGTCCTTTCTG	AGAATTGACCCACCAATCCA

### Hemolymph JH titer

One µl hemolymph was collected from each individual 7-day old bee. For every biological sample, 3 µl hemolymph was pooled randomly from three individuals of the same treatment group. Each biological sample was placed in 500 µl hexane and stored at −80°C prior to analysis. The gas chromatography/mass spectrometry (GC–MS) method of Bergot et al. [104] as modified by Amdam et al. [105] was used to titer JH. Samples were eluted through aluminum oxide columns successively with hexane, 10% ethyl ether–hexane and 30% ethyl ether–hexane. Samples were subjected to a second series of aluminum oxide elutions (30% ethyl ether-hexane then 50% ethyl-acetate–hexane) after derivatization with methyl-d alcohol (Sigma-Aldrich, St Louis, MO, USA) and trifluoroacetic acid (Sigma-Aldrich, St Louis, MO, USA). Purified samples were analyzed on an HP 7890A Series GC (Agilent Technologies, Santa Clara, CA, USA) equipped with a 30 m×0.25 mm Zebron ZB-WAX column (Phenomenex, Torrence, CA, USA) and coupled to an HP 5975C inert mass selective detector. Helium was the carrier gas. MS analysis occurred in the SIM mode, monitoring at m/z 76 and 225 to ensure specificity for the d_3_-methoxyhydrin derivative of JHIII. Total abundance was quantified against a standard curve of derivatized JHIII. The assay's detection limit is 1 pg.

### Bees for testing of sensory sensitivity, starvation resistance, metabolic physiology, and gene expression

Newly emerged bees were obtained from nine wild-type colonies and injected with dsRNA, following the same protocol described above. Approximately 200 bees were injected per treatment group, and thereafter introduced into three nucleus hives containing four frames of honey, pollen and brood, one queen, and a background population of about 5,000 wild-type bees per colony.

### Gustatory responsiveness

The 7-day old treated bees were collected, briefly cold anesthetized then fastened into a metal holder allowing only head mobility [57]. After 1 h, gustatory responsiveness was tested using the proboscis extension response (PER) [11]. The investigator was blind to the treatment identity of the bees. Each worker was tested by touching both antennae with a droplet of H_2_O followed by a concentration series of 0.1, 0.3, 1, 3, 10, 30% sucrose, with a 10 min interstimulus interval. A PER was noted if a bee fully extended its proboscis when a drop of water or sucrose was touched to each antenna. The sum of elicited PERs provided a gustatory response score (GRS) ranging between 0 (no response) and 7 (response to all solutions including H_2_O) [106].

### Starvation resistance

After GRS was determined, the tested bees were kept in their holders. The holders and bees were placed in an incubator set at 34°C and 80% HR. The bees were left unfed and the number of survivors was noted every 3 h for 3 days for survival analysis.

### Circulating carbohydrates in hemolymph and lipid reserves in fat body

A separate set of bees were collected from the three nucleus hives for examining two major circulating carbohydrates, glucose and trehalose, and fat body lipid content.

Carbohydrate content was examined in 1 µl hemolymph sample collected from each individual bee at 7-days post-emergence. Glucose titer was measured using an enzymatic reagent assay (Sigma-Aldrich, St Louis, MO, USA), run for 15 min at 26°C. Using a spectrophotometer (Bio-Rad xMARK Microplate spectrophotometer), A_340_ was measured to determine total glucose in each sample. To measure trehalose [107], the enzyme trehalase (Sigma-Aldrich, St Louis, MO, USA) was added to the hemolymph sample (final concentration is 0.05 units/ml) and the resulting solution was kept at 37°C overnight. A second reading of A_340_ was subsequently taken and the amount of glucose produced from trehalose was calculated by subtracting the amount in the first reading from that in the final reading. The amount of trehalose was calculated using an equation: trehalose (µg) = Glucose (µg)×342.3/(180.2×2). Three replicates were tested for each sample. Final concentration was determined by reference to standard curves.

Fat bodies were collected as well as the hemolymph, and flash-frozen in liquid nitrogen for measuring abdominal lipid content. Each abdomen without digestive tract and sting apparatus, was freeze-dried, homogenized in a 2∶1 chloroform∶methanol solution and dried down to final volume 200 µl. A lipid assay was performed using 100 µl of each sample, following the protocol of Toth et al. [15]. A_525_ was measured and absorbance readings were converted to mg using a curve generated from a cholesterol standard mix. Three replicates were performed for each sample.

### Responses in associated gene networks

Fat bodies and whole heads were collected from another new set of 7-day old marked bees in the same nucleus hives which provided bees for detecting sensory sensitivity, starvation resistance and metabolic physiology. The samples were flash-frozen in liquid nitrogen and stored in a −80°C freezer until use. The same protocols for RNA extraction, reverse transcription and real-time PCR were used which we described above. Primers are listed in table 1.

### *AKH* gene cloning

Total RNA isolated from whole worker heads was used for cloning *AKH*. Total RNA was treated with DNaseI (Invitrogen, Carlsbad, CA, USA) and 5′ and 3′ RACE experiments were performed using the GeneRacer Kit (Invitrogen, Carlsbad, CA, USA) according to the manufacturer's instructions. For the 5′ RACE, a degenerate primer was used in combination with the primer supplied with the kit. For 3′ RACE, a forward primer was used in combination with the 3′RACE primers supplied with the kit. The PCR products were cloned into a T-easy vector (Promega, Madison, WI, USA) following the instructions. Several clones were randomly picked and verified by sequencing. Subsequent to sequence analysis, full-length cDNA of *AKH* was amplified and re-verified by sequencing.

### Statistics

Gene expression data were Log transformed to approximate normality, as verified by Bartlett and Levene's homogeneity test. A full factorial ANOVA was used to test overall effects of treatment on groups, and Fisher LSD (Least Significant Difference) tests were used for most post-hoc comparisons. A student's t-test was used to detect the knockdown effects on JH titer between *vg* or *usp* single knockdowns and *gfp* controls. For the survival analysis, the frequencies of dead bees vs. total number of bees were analyzed with a Chi-square test that contrasted groups receiving different treatment bees. Comparisons of life spans were conducted with the Cox's F survival test. Analyses were performed with STATISTICA 6.0 (StatSoft).

## Supporting Information

Figure S1 Nucleotide and deduced amino acid sequence of *Apis mellifera AKH* precursor cDNA (GenBank: JN983824). The *AKH* gene consists of three exons and is organized similarly to AKH precursors of other species [108]. A signal peptide is followed by a single mature AKH peptide (in boldface with grey shading), followed by the glycine required for canonical amidation and dibasic cleavage signals (GKR). After the stop codon, there are potential polyadenylation signals (AATAAA, AATAT and ATTTT) and an 11-nucleotide poly (A) tail. Conserved cysteines are shaded in grey. Putative polyadenylation signal homologies are underlined. The *A. mellifera* AKH peptide is nearly identical to the *Tribalism* AKH1 peptide sequence QLNFSTG(D)W-amide [71], [72], differing at the 5′ end from the predicted gene GB30028-RA (Genbank).
(PPT)

## References

[1] Seeley TD (1982). Adaptive Significance of the Age Polyethism Schedule in Honeybee Colonies.. Behavioral Ecology and Sociobiology.

[2] Behrends A, Scheiner R, Baker N, Amdam GV (2007). Cognitive aging is linked to social role in honey bees (*Apis mellifera*).. Exp Gerontol.

[3] Robinson GE (1985). Effects of a juvenile hormone analogue on honey bee foraging behaviour and alarm pheromone production.. J Insect Physiol.

[4] Jaycox ER, Skowronek W, Guynn G (1974). Behavioral changes in worker honey bees (*Apis mellifera*) induced by injections of a juvenile hormone mimic.. Ann Entomol Soc Am.

[5] Huang ZY, Robinson GE, Yaoi S, Strambi C, Strambi A (1991). Hormonal regulation of behavioural development in the honey bee is based on changes in the rate of juvenile hormone biosynthesis.. Journal of Insect Physiology.

[6] Ament SA, Corona M, Pollock HS, Robinson GE (2008). Insulin signaling is involved in the regulation of worker division of labor in honey bee colonies.. Proc Natl Acad Sci U S A.

[7] Ament SA, Velarde RA, Kolodkin MH, Moyse D, Robinson GE (2011). Neuropeptide Y-like signalling and nutritionally mediated gene expression and behaviour in the honey bee.. Insect Mol Biol.

[8] Wang Y, Mutti NS, Ihle KE, Siegel A, Dolezal AG (2010). Down-regulation of honey bee IRS gene biases behavior toward food rich in protein.. PLoS Genet.

[9] Pankiw T, Page RE (2000). Response thresholds to sucrose predict foraging division of labor in honeybees.. Behav Ecol Sociobiol.

[10] Pankiw T, Nelson M, Page RE, Fondrk MK (2004). The communal crop: modulation of sucrose response thresholds of pre-foraging honey bees with incoming nectar quality.. Behav Ecol Sociobiol.

[11] Scheiner R, Page RE, Erber J (2001). The effects of genotype, foraging role, and sucrose responsiveness on the tactile learning performance of honey bees (*Apis mellifera* L.).. Neurobiol Learn Mem.

[12] Ben-Shahar Y, Robinson GE (2001). Satiation differentially affects performance in a learning assay by nurse and forager honey bees.. J Comp Physiol A Neuroethol Sens Neural Behav Physiol.

[13] Fluri P, Bogdanov S, Eder K, Rembold H (1987). Age dependence of fat body protein in summer and winter bees (*Apis mellifera*).. Chemistry and Biology of Social Insects.

[14] Haydak MH (1957). Changes with age in the appearance of some internal organs of the honeybee.. Bee World.

[15] Toth AL, Robinson GE (2005). Worker nutrition and division of labour in honeybees.. Anim Behav.

[16] Amdam GV, Omholt SW (2003). The hive bee to forager transition in honeybee colonies: the double repressor hypothesis.. J Theor Biol.

[17] Toth AL, Kantarovich S, Meisel AF, Robinson GE (2005). Nutritional status influences socially regulated foraging ontogeny in honey bees.. J Exp Biol.

[18] Ament SA, Chan QW, Wheeler MM, Nixon SE, Johnson SP (2011). Mechanisms of stable lipid loss in a social insect.. Journal of Experimental Biology.

[19] Wang Y, Amdam GV, Rueppell O, Wallrichs MA, Fondrk MK (2009). PDK1 and HR46 gene homologs tie social behavior to ovary signals.. PLoS ONE.

[20] Wick KL, Liu F (2001). A new molecular target of insulin action: regulating the pivotal PDK1.. Curr Drug Targets Immune Endocr Metabol Disord.

[21] Hunt GJ, Amdam GV, Schlipalius D, Emore C, Sardesai N (2007). Behavioral genomics of honeybee foraging and nest defense.. Naturwissenschaften.

[22] Tobe SS (1985). Structure and regulation of the corpus allatum.. Adv Insect Physiol.

[23] Jaycox ER (1976). Behavioral Changes in Worker Honey Bees (*Apis mellifera* L.) after Injection with Synthetic Juvenile Hormone (Hymenoptera: Apidae).. Journal of the Kansas Entomological Society.

[24] Chapman RF (1998). The Inscects: Structure and Function.

[25] Engels W (1974). Occurrence and significance of vitellogenins in female castes of social hymenoptera.. Am Zool.

[26] Amdam GV, Simões ZLP, Hagen A, Norberg K, Schrøder K (2004). Hormonal control of the yolk precursor vitellogenin regulates immune function and longevity in honeybees.. Exp Gerontol.

[27] Seehuus SC, Norberg K, Gimsa U, Krekling T, Amdam GV (2006). Reproductive protein protects functionally sterile honey bee workers from oxidative stress.. Proc Natl Acad Sci U S A.

[28] Amdam GV, Norberg K, Hagen A, Omholt SW (2003). Social exploitation of vitellogenin.. Proc Natl Acad Sci U S A.

[29] Guidugli KR, Nascimento AM, Amdam GV, Barchuk AR, Omholt SW (2005). Vitellogenin regulates hormonal dynamics in the worker caste of a eusocial insect.. FEBS Letters.

[30] Amdam GV, Norberg K, Page RE, Erber J, Scheiner R (2006). Downregulation of vitellogenin gene activity increases the gustatory responsiveness of honey bee workers (*Apis mellifera*).. Behav Brain Res.

[31] Nelson CM, Ihle K, Amdam GV, Fondrk MK, Page RE (2007). The gene *vitellogenin* has multiple coordinating effects on social organization.. PLoS Biol.

[32] Gulia-Nuss M, Robertson AE, Brown MR, Strand MR (2011). Insulin-like peptides and the target of rapamycin pathway coordinately regulate blood digestion and egg maturation in the mosquito Aedes aegypti.. PLoS ONE.

[33] Shen L, Xing L, Yang Y, Gao Q (2007). Sequence analysis of functional Apisimin-2 cDNA from royal jelly of Chinese honeybee and its expression in Escherichia coli.. Asia Pac J Clin Nutr.

[34] Jia K, Chen D, Riddle DL (2004). The TOR pathway interacts with the insulin signaling pathway to regulate *C. elegans* larval development, metabolism and life span.. Development.

[35] Richard DS, Rybczynski R, Wilson TG, Wang Y, Wayne ML (2005). Insulin signaling is necessary for vitellogenesis in Drosophila melanogaster independent of the roles of juvenile hormone and ecdysteroids: female sterility of the chico(1) insulin signaling mutation is autonomous to the ovary.. J Insect Physiol.

[36] Tu MP, Yin CM, Tatar M (2005). Mutations in insulin signaling pathway alter juvenile hormone synthesis in Drosophila melanogaster.. Gen Comp Endocrinol.

[37] Flatt T, Tu MP, Tatar M (2005). Hormonal pleiotropy and the juvenile hormone regulation of Drosophila development and life history.. Bioessays.

[38] Bitondi MMG, Simões ZLP (1996). The relationship between level of pollen in the diet, vitellogenin and juvenile hormone titres in Africanized *Apis mellifera* workers.. J Apic Res.

[39] Hansen IA, Attardo GM, Park JH, Peng Q, Raikhel AS (2004). Target of rapamycin-mediated amino acid signaling in mosquito anautogeny.. Proc Natl Acad Sci U S A.

[40] Patel A, Fondrk MK, Kaftanoglu O, Emore C, Hunt G (2007). The making of a queen: TOR pathway governs diphenic caste development.. PLoS ONE.

[41] Neufeld TP (2004). Genetic analysis of TOR signaling in Drosophila.. Curr Top Microbiol Immunol.

[42] Mutti NS, Dolezal AG, Wolschin F, Mutti JS, Gill KS (2011). IIS and TOR nutrient-signaling pathways act via juvenile hormone to influence honey bee caste fate.. Journal of Experimental Biology.

[43] Ament SA, Wang Y, Robinson GE (2010). Nutritional regulation of division of labor in honey bees: toward a systems biology perspective.. Wiley Interdiscip Rev Syst Biol Med.

[44] Sullivan JP, Fahrbach SE, Harrison JF, Capaldi EA, Fewell JH (2003). Juvenile hormone and division of labor in honey bee colonies: effects of allatectomy on flight behavior and metabolism.. J Exp Biol.

[45] Piulachs MD, Guidugli KR, Barchuk AR, Cruz J, Simões ZLP (2003). The vitellogenin of the honey bee, *Apis mellifera*: structural analysis of the cDNA and expression studies.. Insect Biochem Mol Biol.

[46] Jones G, Sharp PA (1997). Ultraspiracle: an invertebrate nuclear receptor for juvenile hormones.. Proc Natl Acad Sci U S A.

[47] Miura K, Oda M, Makita S, Chinzei Y (2005). Characterization of the Drosophila Methoprene -tolerant gene product. Juvenile hormone binding and ligand-dependent gene regulation.. Febs J.

[48] Riddiford LM (2008). Juvenile hormone action: a 2007 perspective.. J Insect Physiol.

[49] Barchuk AR, Maleszka R, Simoes ZLP (2004). *Apis mellifera* ultraspiracle: cDNA sequence and rapid up-regulation by juvenile hormone.. Insect Mol Biol.

[50] Barchuk AR, Figueiredo VL, Simoes ZL (2008). Downregulation of ultraspiracle gene expression delays pupal development in honeybees.. J Insect Physiol.

[51] Elias-Neto M, Soares MP, Simoes ZL, Hartfelder K, Bitondi MM (2010). Developmental characterization, function and regulation of a Laccase2 encoding gene in the honey bee, *Apis mellifera* (Hymenoptera, Apinae).. Insect Biochem Mol Biol.

[52] Mackert A, Nascimento AM, Bitondi MMG, Hartfelder K, Simões ZLP (2008). Identification of a juvenile hormone esterase-like gene in the honey bee, *Apis mellifera* L. — Expression analysis and functional assays.. Comp Biochem Physiol B.

[53] Mutti NS, Wang Y, Kaftanoglu O, Amdam GV (2011). Honey bee PTEN—‘description, developmental knockdown, and tissue-specific expression of splice-variants correlated with alternative social phenotypes.. PLoS ONE.

[54] Nilsen K, Ihle KE, Frederick K, Fondrk MK, Smedal B (2011). In Honeybee Fat Body, Insulin-1 Like Peptide Genes Respond Differently to Manipulation of Social Behavioral Physiology.. Journal of Experimental Biology.

[55] Jarosch A, Moritz RFA (2011). SystemicRNA-interferenceinthehoneybee *Apis mellifera*: Tissue dependent uptake of fluorescent siRNA after intra-abdominal application observed by laser-scanning microscopy.. J Insect Physiol.

[56] Pankiw T, Page RE (2003). Effect of pheromones, hormones, and handling on sucrose response thresholds of honey bees (*Apis mellifera* L.).. J Comp Physiol A Neuroethol Sens Neural Behav Physiol.

[57] Bitterman ME, Menzel R, Fietz A, Schäfer S (1983). Classical conditioning of proboscis extension in honeybees (*Apis mellifera*).. J Comp Physiol.

[58] Page RE, Erber J, Fondrk MK (1998). The effect of genotype on response thresholds to sucrose and foraging behavior of honey bees (*Apis mellifera* L.).. J Comp Physiol [A].

[59] Scheiner R, Page RE, Erber J (2001). Responsiveness to sucrose affects tactile and olfactory learning in preforaging honey bees of two genetic strains.. Behav Brain Res.

[60] Ballard JW, Melvin RG, Simpson SJ (2008). Starvation resistance is positively correlated with body lipid proportion in five wild caught Drosophila simulans populations.. J Insect Physiol.

[61] Woodring J, Boulden M, Das S, Gade G (1993). Studies on blood sugar homeostasis in the honeybee (*Apis mellifera*, L.).. Journal of Insect Physiology.

[62] Candy DJ, Becker A, Wegener G (1997). Coordination and integration of metabolism in insect flight.. Comparative Biochemistry and Physiology B-Biochemistry & Molecular Biology.

[63] Giannakou ME, Goss M, Junger MA, Hafen E, Leevers SJ (2004). Long-lived Drosophila with overexpressed dFOXO in adult fat body.. Science.

[64] Kaun KR, Riedl CA, Chakaborty-Chatterjee M, Belay AT, Douglas SJ (2007). Natural variation in food acquisition mediated via a Drosophila cGMP-dependent protein kinase.. J Exp Biol.

[65] Scheiner R, Sokolowski MB, Erber J (2004). Activity of cGMP-dependent protein kinase (PKG) affects sucrose responsiveness and habituation in Drosophila melanogaster.. Learn Mem.

[66] Ben-Shahar Y (2005). The foraging gene, behavioral plasticity, and honeybee division of labor.. J Comp Physiol A Neuroethol Sens Neural Behav Physiol.

[67] Ben-Shahar Y, Leung HT, Pak WL, Sokolowski MB, Robinson GE (2003). cGMP-dependent changes in phototaxis: a possible role for the foraging gene in honey bee division of labor.. J Exp Biol.

[68] Wang S, Shiva S, Poczatek MH, Darley-Usmar V, Murphy-Ullrich JE (2002). Nitric oxide and cGMP-dependent protein kinase regulation of glucose-mediated thrombospondin 1-dependent transforming growth factor-beta activation in mesangial cells.. J Biol Chem.

[69] Zanetti M, Barazzoni R, Stebel M, Roder E, Biolo G (2005). Dysregulation of the endothelial nitric oxide synthase-soluble guanylate cyclase pathway is normalized by insulin in the aorta of diabetic rat.. Atherosclerosis.

[70] Hummon AB, Richmond TA, Verleyen P, Baggerman G, Huybrechts J (2006). From the genome to the proteome: uncovering peptides in the Apis brain.. Science.

[71] Li B, Predel R, Neupert S, Hauser F, Tanaka Y (2008). Genomics, transcriptomics, and peptidomics of neuropeptides and protein hormones in the red flour beetle Tribolium castaneum.. Genome Res.

[72] Roller L, Yamanaka N, Watanabe K, Daubnerova I, Zitnan D (2008). The unique evolution of neuropeptide genes in the silkworm Bombyx mori.. Insect Biochem Mol Biol.

[73] Persani L, Calebiro D, Cordella D, Weber G, Gelmini G (2010). Genetics and phenomics of hypothyroidism due to TSH resistance.. Molecular and Cellular Endocrinology.

[74] Yoshida H, Yokode M, Yamamoto A, Masaki R, Murayama T (1999). Compensated endocytosis of LDL by hamster cells co-expressing the two distinct mutant LDL receptors defective in endocytosis and ligand binding.. Journal of Lipid Research.

[75] Billas IM, Moras D (2005). Ligand-binding pocket of the ecdysone receptor.. Vitam Horm.

[76] Hartfelder K, Bitondi MMG, Santana WC, Simões ZLP (2002). Ecdysteroid titer and reproduction in queens and workers of the honey bee and of a stingless bee: loss of ecdysteroid function at increasing levels of sociality?. Insect Biochem Mol Biol.

[77] Velarde RA, Robinson GE, Fahrbach SE (2009). Coordinated responses to developmental hormones in the Kenyon cells of the adult worker honey bee brain (*Apis mellifera* L.).. J Insect Physiol.

[78] Huang ZY, Robinson GE, Borst DW (1994). Physiological correlates of division of labor among similarly aged honey bees.. J Comp Physiol [A].

[79] Ben-Shahar Y, Robichon A, Sokolowski MB, Robinson GE (2002). Influence of gene action across different time scales on behavior.. Science.

[80] Rulifson EJ, Kim SK, Nusse R (2002). Ablation of insulin-producing neurons in flies: growth and diabetic phenotypes.. Science.

[81] Belgacem YH, Martin JR (2006). Disruption of insulin pathways alters trehalose level and abolishes sexual dimorphism in locomotor activity in Drosophila.. J Neurobiol.

[82] Broughton S, Alic N, Slack C, Bass T, Ikeya T (2008). Reduction of DILP2 in Drosophila triages a metabolic phenotype from lifespan revealing redundancy and compensation among DILPs.. PLoS ONE.

[83] Sheng ZT, Xu JJ, Bai H, Zhu F, Palli SR (2011). Juvenile Hormone Regulates Vitellogenin Gene Expression through Insulin-like Peptide Signaling Pathway in the Red Flour Beetle, Tribolium castaneum.. Journal of Biological Chemistry.

[84] Bharucha KN, Tarr P, Zipursky SL (2008). A glucagon-like endocrine pathway in Drosophila modulates both lipid and carbohydrate homeostasis.. J Exp Biol.

[85] Gronke S, Muller G, Hirsch J, Fellert S, Andreou A (2007). Dual lipolytic control of body fat storage and mobilization in Drosophila.. PLoS Biol.

[86] Musselman LP, Fink JL, Narzinski K, Ramachandran PV, Hathiramani SS (2011). A high-sugar diet produces obesity and insulin resistance in wild-type Drosophila.. Dis Model Mech.

[87] Ziegler R, Isoe J, Moore W, Riehle MA, Wells MA (2010). The putative AKH receptor of the tobacco hornworm, Manduca sexta, and its expression.. J Insect Sci.

[88] Kaun KR, Chakaborty-Chatterjee M, Sokolowski MB (2008). Natural variation in plasticity of glucose homeostasis and food intake.. J Exp Biol.

[89] Ninomiya Y, Shigemura N, Yasumatsu K, Ohta R, Sugimoto K (2002). Leptin and sweet taste.. Vitam Horm.

[90] Friedman JM, Halaas JL (1998). Leptin and the regulation of body weight in mammals.. Nature.

[91] Ahima RS, Osei SY (2001). Molecular regulation of eating behavior: new insights and prospects for therapeutic strategies.. Trends Mol Med.

[92] Giza BK, Scott TR (1987). Blood glucose level affects perceived sweetness intensity in rats.. Physiol Behav.

[93] Wu Q (2005). FUNCTIONAL ANALYSES OF THE DROSOPHILA INSULIN- AND NEUROPEPTIDE Y-LIKE SIGNALING SYSTEMS: REGULATION OF FEEDING, RISK-AVERSE AND SOCIAL BEHAVIOR..

[94] Drewnowski A, Brunzell JD, Sande K, Iverius PH, Greenwood MR (1985). Sweet tooth reconsidered: taste responsiveness in human obesity.. Physiol Behav.

[95] Bartoshuk LM, Duffy VB, Hayes JE, Moskowitz HR, Snyder DJ (2006). Psychophysics of sweet and fat perception in obesity: problems, solutions and new perspectives.. Philosophical Transactions of the Royal Society B-Biological Sciences.

[96] Amdam GV, Simoes ZL, Guidugli KR, Norberg K, Omholt SW (2003). Disruption of vitellogenin gene function in adult honeybees by intra-abdominal injection of double-stranded RNA.. BMC Biotechnol.

[97] Amdam GV, Nilsen KA, Norberg K, Fondrk MK, Hartfelder K (2007). Variation in endocrine signaling underlies variation in social life history.. Am Nat.

[98] Bieke Scharlaken DCdG, Goossens Karen, Brunain Marleen, Peelman LucJ, Jacobs FransJ (2008). Reference gene selection for insect expression studies using quantitative real-time PCR: The head of the honeybee, *Apis mellifera*, after a bacterial challenge.. Journal of insect Science.

[99] Anete Pedro Lourenço AM, Alexandre dos Santos Cristino and Zilá Luz Paulino Simões (2008). Validation of reference genes for gene expression studies in the honey bee, *Apis mellifera*, by quantitative real-time RT-PCR.. Apidologie.

[100] Amdam GV, Norberg K, Fondrk MK, Page RE (2004). Reproductive ground plan may mediate colony-level selection effects on individual foraging behavior in honey bees.. Proc Natl Acad Sci U S A.

[101] de Azevedo SV, Hartfelder K (2008). The insulin signaling pathway in honey bee (*Apis mellifera*) caste development - differential expression of insulin-like peptides and insulin receptors in queen and worker larvae.. J Insect Physiol.

[102] Pfaffl MW (2001). A new mathematical model for relative quantification in real-time RT-PCR.. Nucleic Acids Res.

[103] Vandesompele J, De Paepe A, Speleman F (2002). Elimination of primer-dimer artifacts and genomic coamplification using a two-step SYBR green I real-time RT-PCR.. Anal Biochem.

[104] Bergot BJ, Schooley DA, Dekort CAD (1981). Identification of Jh-Iii as the Principal Juvenile-Hormone in Locusta-Migratoria.. Experientia.

[105] Amdam GV, Page RE, Fondrk MK, Brent CS (2010). Hormone response to bidirectional selection on social behavior.. Evolution & Development.

[106] Scheiner R (2004). Responsiveness to sucrose and habituation of the proboscis extension response in honey bees.. J Comp Physiol A.

[107] Broughton SJ, Piper MDW, Ikeya T, Bass TM, Jacobson J (2005). Longer lifespan, altered metabolism, and stress resistance in Drosophila from ablation of cells making insulin-like ligands.. Proc Natl Acad Sci USA.

[108] Gade G, Hoffmann KH, Spring JH (1997). Hormonal regulation in insects: facts, gaps, and future directions.. Physiol Rev.

