# Supplementary figures and images for: Gustatory Perception and Fat Body Energy Metabolism Are Jointly Affected by Vitellogenin and Juvenile Hormone in Honey Bees
Source: PLoS Genet. 2012 Jun 28;8(6):e1002779. doi: 10.1371/journal.pgen.1002779 (PMC3386229; doi:10.1371/journal.pgen.1002779)

## Slide 1
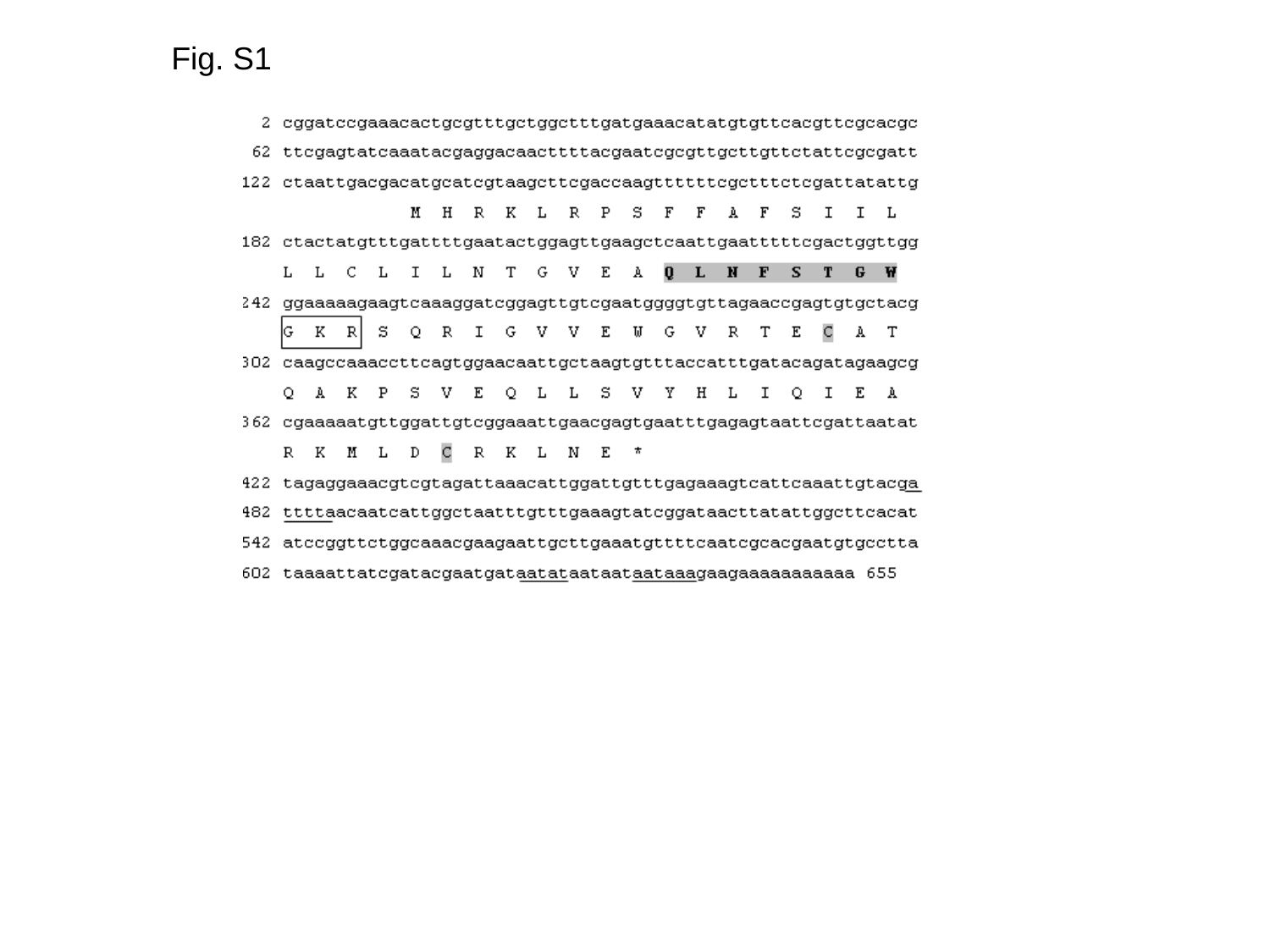

Fig. S1

Supplement: Figure S1 — Nucleotide and deduced amino acid sequence of Apis mellifera AKH precursor cDNA (GenBank: JN983824). The AKH gene consists of three exons and is organized similarly to AKH precursors of other species [108]. A signal peptide is followed by a single mature AKH peptide (in boldface with grey shading), followed by the glycine required for canonical amidation and dibasic cleavage signals (GKR). After the stop codon, there are potential polyadenylation signals (AATAAA, AATAT and ATTTT) and an 11-nucleotide poly (A) tail. Conserved cysteines are shaded in grey. Putative polyadenylation signal homologies are underlined. The A. mellifera AKH peptide is nearly identical to the Tribalism AKH1 peptide sequence QLNFSTG(D)W-amide [71], [72], differing at the 5′ end from the predicted gene GB30028-RA (Genbank). (PPT) [file pgen.1002779.s001.ppt]
